# Cytokine-Mediated Bone Destruction in Rheumatoid Arthritis

**DOI:** 10.1155/2014/263625

**Published:** 2014-09-10

**Authors:** Seung Min Jung, Kyoung Woon Kim, Chul-Woo Yang, Sung-Hwan Park, Ji Hyeon Ju

**Affiliations:** ^1^Division of Rheumatology, Department of Internal Medicine, Seoul St. Mary's Hospital, College of Medicine, The Catholic University of Korea, 222 Banpo-Daero, Seocho-Gu, Seoul 137-701, Republic of Korea; ^2^Convergent Research Consortium for Immunologic Disease, Seoul St. Mary's Hospital, College of Medicine, The Catholic University of Korea, Seoul, Republic of Korea; ^3^Division of Nephrology, Department of Internal Medicine, Seoul St. Mary's Hospital, College of Medicine, The Catholic University of Korea, Seoul, Republic of Korea

## Abstract

Bone homeostasis, which involves formation and resorption, is an important process for maintaining adequate bone mass in humans. Rheumatoid arthritis (RA) is an autoimmune disease characterized by inflammation and bone loss, leading to joint destruction and deformity, and is a representative disease of disrupted bone homeostasis. The bone loss and joint destruction are mediated by immunological insults by proinflammatory cytokines and various immune cells. The connection between bone and immunity has been intensely studied and comprises the emerging field of osteoimmunology. Osteoimmunology is an interdisciplinary science investigating the interplay between the skeletal and the immune systems. The main contributors in osteoimmunology are the bone effector cells, such as osteoclasts or osteoblasts, and the immune cells, particularly lymphocytes and monocytes. Physiologically, osteoclasts originate from immune cells, and immune cells regulate osteoblasts and vice versa. Pathological conditions such as RA might affect these interactions, thereby altering bone homeostasis, resulting in the unfavorable outcome of bone destruction. In this review, we describe the osteoclastogenic roles of the proinflammatory cytokines and immune cells that are important in the pathophysiology of RA.

## 1. Introduction

Rheumatoid arthritis (RA) is a devastating autoimmune disease characterized by progressive bone destruction. Under physiological conditions, bone remodeling occurs continually, as a coordinated process that results in the formation and degradation of bone. This process is a balance between bone formation, which is mediated by osteoblasts, and bone resorption, which is regulated by osteoclasts, and ensures bone homeostasis. In pathological conditions such as RA, bone homeostasis is disrupted, resulting in uncoordinated osteoclast formation.

**Figure 1:**
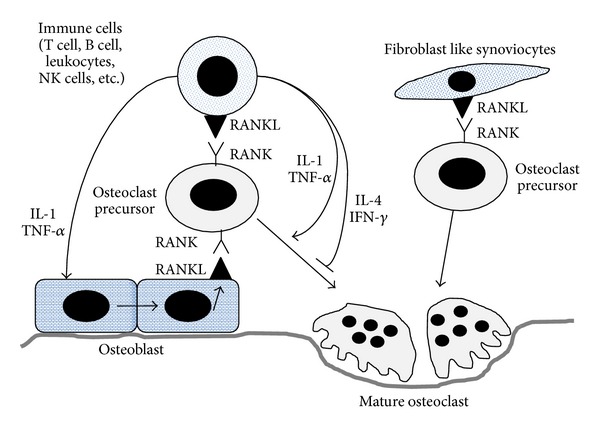
Osteoblast-derived RANKL binds to RANK on monocytes to differentiate them into mature osteoclasts. Osteoblast-derived RANKL plays important role in generating osteoclast in physiological condition. However, immune cell and FLS-derived RANKL play pathogenic role in RA. Proinflammatory cytokines such as IL-1 and TNFα effectively stimulate osteoblast to express RANKL. FLS-derived RANKL enhances osteoclastogenesis in RA joints. RANK: receptor activator of the nuclear factor kappa B; RANKL: receptor activator of the nuclear factor kappa B ligand; FLS: fibroblast like synoviocyte; RA: rheumatoid arthritis; IL-1: Interleukin-1; TNFα: tumor necrosis factor-alpha.

Osteoclasts are generated from precursor cells that are usually of the monocyte-macrophage lineage. Interactions between receptor activator of the nuclear factor kappa B (RANK) and its ligand (RANKL) are essential in osteoclastogenesis. RANK on monocyte binds to RANKL, initiating osteoclast differentiation. Under physiological conditions, the main source of RANKL is osteoblasts. However, immune cells and fibroblast-like synoviocytes (FLS) are the main source of RANKL in pathological conditions such as arthritic RA joints (Figure 1). Several systemic and local factors influence the process of osteoclastogenesis. In RA, excessive activation of the immune system could affect the formation and function of osteoclasts. Proinflammatory cytokines tend to be osteoclastogenic; however, the opposite has also been observed [1]. In our literature review, proinflammatory cytokines such as interleukin (IL)-1, IL-6, IL-8, IL-11, IL-17, and tumor necrosis factor (TNF)-α were frequently reported to be osteoclastogenic, and IL-4, IL-10, IL-13, IL-18, interferon (IFN)-*γ*, and IFN-*β* were anti-osteoclastogenic. T cell subpopulations have been studied for their contribution to osteoimmunology. T helper 17 cells (Th17 cells), a specific subtype of T helper cells that produce IL-17 and RANKL, were reported to be osteoclastogenic, whereas the classical Th1 and Th2 cells were generally reported to be anti-osteoclastogenic through their production of IFN-*γ* (Th1) and IL-4 (Th2) [2, 3].

We could not draw uniform conclusions about the various factors involved in osteoclastogenesis. Some proinflammatory cytokines, such as IL-7, IL-12, IL-23, and TGF-*β*, possess dual osteoclastogenic and anti-osteoclastogenic properties. Their net effect depends on the specific pathophysiological conditions in* in vivo* models, whereas it depends on the developmental stage of the osteoclasts [4–6] in* in vitro* experiments. The determination of their exact role in the bone microenvironment is even more difficult because these cytokines can have synergistic or antagonistic effects on osteoclasts [7–11].

**Figure 2:**
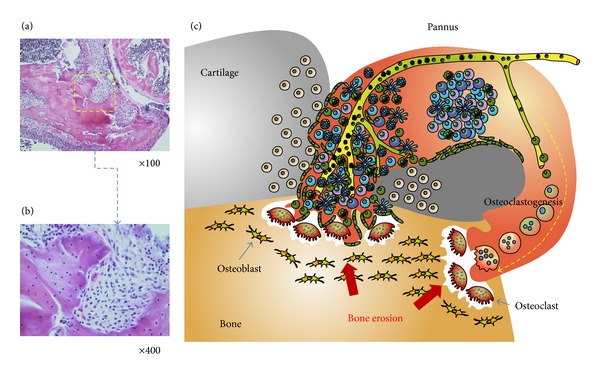
(a) Bone is destroyed by a proliferative and invasive synovium, which is called pannus. It originates from adjacent synovial tissue and invades the cartilages and bones. (b) Magnified view of the pannus-bone interface. The pannus-bone interface is lined with mature osteoclasts (arrows). Various inflammatory cells and stromal cells comprise the invading pannus. (c) Schematic depiction of the pannus-cartilage-bone structure. Inflammatory cells such as B cells, T cells, macrophages, monocytes, and fibroblast-like synoviocytes accumulate in the pannus. For metabolic support, intensive angiogenesis is usually followed. Excessive provision of RANKL from the accumulated cells in the pannus enhances osteoclastogenesis, resulting in the erosion of bone at the pannus-bone interface.

The joint structure is invaded and the bone is destroyed by the pannus, which contains a massive infiltration of immune cells, proliferative vessels, and increased numbers of osteoclasts (Figures 2(a) and 2(b)). These complicated structures are frequently observed in RA at the synovium-bone interface (Figure 2(c)). This review will address immune-mediated bone destruction in two sections. First, the osteoclastogenic role of proinflammatory cytokines will be discussed. In the following section, the osteoclastogenic role of inflammatory cells that play important roles in the pathogenesis of RA will be described.

## 2. Cytokines and Bone: The Osteoclastogenic Effect of Proinflammatory Cytokines

Proinflammatory cytokines promote osteoclastogenesis via RANKL expression. Some researchers have shown that proinflammatory cytokines such as TNF-α, IL-1, and IL-6 are capable of inducing osteoclast differentiation independently of RANKL [12–14]. Others showed that a minimal level of RANKL is essential for TNF-α-induced osteoclastogenesis, revealing that TNF-α alone does not induce osteoclast formation [15]. To clarify this controversy, we adopted a simplified monocellular culture system instead of a co-culture system, which consists of osteoblasts and bone marrow cells [16]. In our experience, permissive levels of RANKL were required for cytokine-associated osteoclastogenesis. IL-1 increased and IL-6 decreased the number of mature osteoclasts in a dose-dependent manner. Treatment with IL-23, IL-17, or TNF-α resulted in various responses according to the exposure time and the cytokine concentration.

The effects of important cytokines on osteoclastogenesis* in vitro* and* in vivo* are summarized in Table 1. Based on laboratory observations, cytokine-targeting therapies were tested in bone resorptive conditions. The results of experimental and clinical trials are presented in Table 2.

### 2.1. TNF-α

TNF-α has received attention from immunologists and rheumatologists because several TNF-α inhibitors show enormous pharmaceutical success in treating RA. TNF-α is produced by activated T cells and is involved in inflammation- and cancer-induced bone loss [17]. Treatment with TNF-α inhibitors results in decreased inflammation and bone protection in RA patients [18].* In vivo* blockade of TNF-α reduces bone resorption in postmenopausal osteoporosis [19]. Thus, TNF-α is regarded as a major contributor to bone destruction and osteoclast formation.

TNF-α promotes bone destruction by upregulating the production of RANKL and macrophage colony-stimulating factor (M-CSF) from osteoblasts and stromal cells, and by augmenting differentiation into osteoclasts independently of RANK-RANKL signaling [20]. In addition, TNF-α and RANKL synergistically upregulate the expression of RANK [21]. This osteoclastogenic effect of TNF-α is closely associated with other inflammatory cytokines, including IL-1 and M-CSF [22–24]. Although osteoclastogenesis is a more dominant mechanism in the bone erosion of inflammatory disease, osteoblast formation is also affected by TNF-α. TNF-α inhibits osteoblast differentiation primarily through TNF-receptor 1 signaling [25, 26].

### 2.2. IL-1

IL-1, a proinflammatory cytokine, is highly expressed in patients with RA [27]. An earlier study showed a prominent protective effect of IL-1 blockade against structural damage in an arthritis animal model, suggesting a crucial effect of IL-1 on bone metabolism [28]. Animal models with a deficiency of IL-1 signaling present with reduced osteoclastogenesis, leading to significantly increased levels of bone density, trabecular bone mass, and cortical thickness [29, 30]. IL-1 also plays an important role in the bone loss induced by estrogen deficiency; the level of IL-1 increases after menopause and decreases with estrogen replacement [31, 32]. Bone resorption is suppressed by blockade of IL-1 in postmenopausal women [19].

IL-1 induces RANKL to promote osteoclastogenesis through the production of prostaglandin E in periodontal tissue [33, 34]. Furthermore, IL-1 might exert a bone resorptive effect via an alternative pathway independent of the RANK/RANKL signal [35, 36]. IL-1 is essential for TNF-α-induced osteoclastogenesis. Human TNF-α transgenic mice lacking IL-1*β* were protected from systemic bone loss regardless of sustained inflammation [37]. The activation of p38 mitogen-activated proteinase kinase is involved in TNF-α- and IL-1-mediated osteoclastogenesis by upregulating RANKL expression in stromal cells and stimulating osteoclast precursor differentiation [23].

### 2.3. IL-6

Dysregulation of IL-6 is frequently observed in RA patients [38–40]. IL-6 is responsible for synovial inflammation as well as the structural damage of RA. An IL-6 receptor antagonist, a new immunotherapeutic, reduced bone turnover, favoring bone protection in RA patients [41, 42]. IL-6 is also involved in other diseases associated with accelerated bone turnover, such as multiple myeloma and Paget's disease of bone [43].

The previous data indicate the dual functions of IL-6 on bone remodeling. The addition of IL-6 and the soluble IL-6 receptor into bone tissue cultures stimulates bone resorption through increased RANKL expression on osteoblasts [44] via activation of the STAT3 pathway [45]. However, IL-6 exhibits a direct inhibitory effect on RANK signaling in osteoclast progenitor cells in the absence of other supporting cells [6].

*In vivo* studies also suggest that the role of IL-6 varies in a context-dependent manner. IL-6 transgenic mice with a high level of circulating IL-6 exhibited enhanced osteoclastogenesis, leading to impaired skeletal growth at the prepubertal stage [46] but decreased osteoclast formation at the adult stage [46, 47]. Under physiological conditions, IL-6 deficiency resulted in no detectable change in osteoclast number [48]. However, IL-6 knockout mice were protected against ovariectomy-induced bone loss [48]. IL-6 knockout mice with experimental arthritis showed significantly decreased osteoclastogenic activity and impaired osteoclast recruitment to inflammatory sites [49]. These results indicate that IL-6 is associated with bone loss from inflammation and estrogen deprivation. IL-6, along with TGF-*β*, induces the differentiation of naïve T cells into Th17 cells, which are typically osteoclastogenic [50].

### 2.4. IL-17

IL-17 is predominantly expressed by Th17 cells, a specific type of human T helper cells [51]. It is hypothesized that this cytokine plays a crucial role in inflammation and the development of autoimmune diseases, including RA. There is evidence that IL-17 enhances osteoclastogenesis by a RANKL-RANK dependent mechanism. Studies of an arthritis animal model indicate that IL-17 induces the expression of RANKL and proinflammatory cytokines such as IL-1 and TNF-α [52]. These inflammatory mediators (IL-17, IL-1, TNF-α, and RANKL) interact with each other in the progression of RA. IL-17A also upregulates the expression of RANK on osteoclast precursors and increases their sensitivity to RANKL [53]. Similarly, treatment with an IL-17 neutralizing antibody inhibited bone destruction in collagen-induced arthritis [54, 55]. However, the mechanisms of action of IL-17 in bone erosion remain to be determined, particularly in association with other osteoclastogenic cytokines such as IL-1, TNF-α, and RANKL.

### 2.5. IL-23

One of the most important stimuli for IL-17 synthesis is IL-23 produced by activated dendritic cells and macrophages [50]. IL-23 is implicated in inflammatory diseases, in association with IL-17. Accordingly, the IL-23/IL-17 axis plays a critical role in controlling inflammatory bone loss. Recent work suggests that osteoclastogenesis is promoted by IL-23 and inhibited by an anti-IL-23 antibody [56]. By contrast, another study shows the indirect inhibition of osteoclast differentiation by IL-23* in vitro*. Under physiological conditions, IL-23 promotes higher bone mass in long bones by limiting bone resorption near the growth plate* in vivo* [57]. These conflicting data suggest different roles for this cytokine in physiological or inflammatory bone turnover.

## 3. Immune Cells and Bone: The Osteoclastogenic Effect of Inflammatory Cells

**Figure 3:**
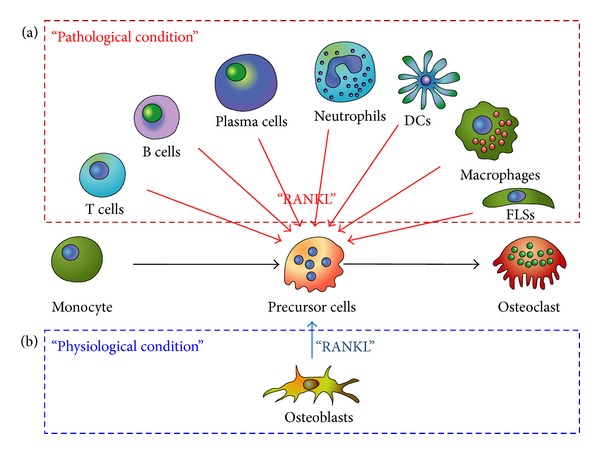
(a) Monocytes are differentiated into mature osteoclasts by the aid of RANKL. In pathological conditions such as inflammation, cancer, and hypermetabolism, various cells extraordinarily provide RANKL to the monocytes, resulting in overweighed osteoclastogenesis. In this condition, the osteoclasts outnumber the osteoblasts, disrupting the bone homeostasis. Bone erosion or osteoporosis is the major outcome of disrupted homeostasis. (b) In normal physiological conditions, a few cells, predominantly osteoblasts, express RANKL. A similar number of osteoclasts and osteoblasts maintain the bone mass by homeostatic equilibrium.

Various immune cells play important roles in the pathogenesis of RA. These cells comprise the rheumatoid synovium that is continuously inflamed and invades adjacent tissue, resulting in joint destruction (Figure 2). Although osteoclasts are the final effectors of bone erosion, osteoclastogenesis is regulated by various cells in the RA synovium. FLS are the main cellular component of the matrix that is involved in bone turnover. Monocytes, T cells, B cells, and neutrophils also infiltrate the RA synovium and interact with each other. These cells vigorously contribute to osteoclast formation under inflammatory conditions by producing osteoclastogenic cytokines or RANKL (Figure 3).

### 3.1. Fibroblast-Like Synoviocytes (FLS)

Under physiological conditions, the synovium secretes synovial fluid and provides mechanical stability to the joint. However, pathological conditions such as RA render the synovium more aggressive. The synovium forms a pannus with inflammatory cells, enabling invasion into the bone [58, 59]. Histopathology demonstrates increased bone resorption at the bone-pannus interface in the joints of patients with RA. Thus, FLS play an active role in the pathogenesis of RA [59].

**Figure 4:**
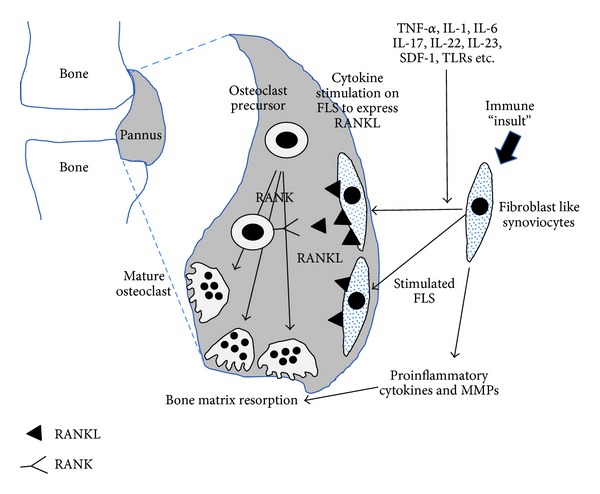
FLS could express RANKL in response to various stimuli. FLS-expressed RANKL enhances osteoclastogenesis and results in bone erosion in RA. Inflammatory and immune stimulation induce the FLS to produce proinflammatory cytokines and matrix metalloproteinase. These cytokines and enzymes aid osteoclasts to destroy the bone matrix.

The bone and cartilage destruction in RA patients is partly mediated by metalloproteinases secreted by activated synoviocytes and chondrocytes [60, 61]. More importantly, bone destruction is further exacerbated by osteoclasts induced by the RA synovium [62, 63]. We reported that RANKL is produced by FLS from RA patients (RA-FLS) and that osteoclasts are formed in cocultures of RA-FLS and human monocytes [64]. Consistent with a previous report [62], this result indicates that RA-FLS have the capability to support osteoclast differentiation. In RA, FLS upregulate the expression of RANKL and osteoclastogenic cytokines. Earlier studies show that RANKL in RA-FLS can be increased by IL-23 [65], IL-22 [66], and SDF-1 [67]. Furthermore, FLS produce osteoclastogenic cytokines such as IL-6 in response to IL-17 and IL-23 [68, 69]. These inflammatory mediators from stimulated RA-FLS act on stromal cells to upregulate RANKL expression and on osteoclast precursor cells to promote differentiation into osteoclasts (Figure 4).

### 3.2. Monocyte and Dendritic Cells

Bloodstream monocytes migrate into inflammatory tissue where they differentiate into resident macrophages and dendritic cells (DCs) [70]. Macrophages and DCs express a variety of inflammatory cytokines involved in the pathogenesis of RA [71].

Synovial macrophages play a central role in rheumatoid inflammation. TNF-α, IL-1, and IL-6 are largely produced by activated macrophages and synovial fibroblasts in the RA synovium [71, 72]. As discussed above, these cytokines directly exert osteoclastogenic effects, either synergistically with RANKL or independently of the RANKL signaling pathway. Moreover, macrophages in the RA synovium also secrete TGF-*β*, IL-21, and IL-23 to differentiate CD4+ T cells into Th17 cells, which are typically referred to as osteoclastogenic T cells.

DC, highly differentiated antigen-presenting cells, interact with T cells and B cells in RA. The physiological function of DC in bone remodeling appears to be modest, as DCs are not frequently observed in bone or the adjacent stroma under normal conditions. By contrast, active lesions of RA and periodontitis retain mature and immature DCs [73–78]. At these sites, DCs contact and interact with T cells to elicit inflammatory processes that involve RANK-RANKL signaling [77]. In multiple myeloma, DCs promote osteoclastogenesis, leading to bone destruction, possibly by activation of RANK-RANKL signaling [79] and the overproduction of IL-17 [80].

DCs can also affect bone metabolism in a more direct manner. Rivollier and colleagues showed that human monocyte-derived DCs transdifferentiate into osteoclasts in the presence of M-CSF and RANKL* in vitro*, suggesting that DCs might directly contribute to osteoclastogenesis [81]. Alnaeeli et al. tested whether the interaction between DCs and T cells supports osteoclast development using an* in vitro* co-culture system of bone marrow-derived CD11c+ DC and CD4+ T cells. Murine CD11c+ DC developed into functional osteoclasts after interactions with CD4+ T cells and stimulation with microbial or protein antigens. Adoptive transfer of DC-derived osteoclasts could induce bone resorption in NOD/SCID mice calvarias* in vivo* [82]. The differentiation of DCs into osteoclasts is frequently reported in the pathogenesis of multiple myeloma [79, 83].

### 3.3. T Cells

T cells are one of the key regulators of synovial inflammation in RA, having both stimulatory and inhibitory roles [71]. T cells can also play a destructive or a protective role in bone metabolism in a context- and subtype-dependent manner.

In the resting state, T cells seem to have a positive effect on bone mineral density, as T cell depletion increased osteoclastogenesis* in vitro* [84] and accelerated bone resorption* in vivo* [85]. T cell-deficient nude mice have significantly higher numbers of osteoclasts and reduced bone density compared to controls [85].

In response to antigenic stimuli, CD4+ T cells differentiate into distinct effector subsets, Th1 and Th2 cells, which are classically defined on the basis of cytokine production profiles [86]. Th1 cells are characterized by the secretion of IFN-*γ*, IL-2, IL-12, TNF-α, and TNF-*β*, and are involved in the elimination of intracellular pathogens [87]. Th2 cells produce IL-4, IL-5, IL-6, IL-9, and IL-13, and are responsible for parasite eradication and allergic disorders [87, 88]. In one comprehensive study, Th1 and Th2 cells were shown to inhibit osteoclastogenesis through IFN-*γ* and IL-4, respectively [89]. However, the bone-preserving effects of Th1 and Th2 cells are not certain, because contradictory responses have been observed in inflammatory conditions. Infection and inflammation could activate T cells to produce osteoclastogenic cytokines such as TNF-α and RANKL. In the pathogenic state, lymphocytes express significantly higher levels of RANKL and have the capacity to induce RANKL-dependent osteoclast differentiation, unlike in healthy conditions [90]. In addition, IFN-*γ* exerts a bone resorptive effect instead of a bone-protective effect in an animal model with ovariectomy, infection, and inflammation [4, 91]. Thus, further research is required to understand the net effect of Th1/Th2 cells in disease states such as RA.

**Figure 5:**
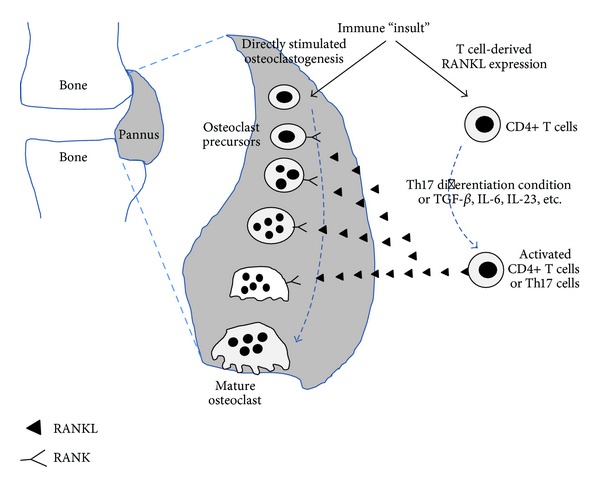
T cells are activated to produce RANKL or osteoclastogenic cytokines by various stimuli. RANKL and activated T cell-cytokines have the potential to induce osteoclastogenesis. With T cells, the outnumbered osteoclasts destroy the bone in RA.

Th17 cells, a more recently characterized subset of CD4+ T cells, have been shown to be more osteoclastogenic. Th17 cells are produced when naïve T cells are activated by TGF-*β* and IL-6 in mice or TGF-*β* and inflammatory cytokines in humans [50, 92]. Th17 cell play a pivotal role in the pathogenesis of RA through the production of Th17 signature cytokines [50]. Since IL-17 is predominantly produced by Th17 cells and is closely associated with osteoclastogenesis, Th17 cells are likely to affect bone metabolism primarily through IL-17. IL-17 directly induces the expression of RANKL from surrounding cells and facilitates the recruitment of inflammatory cells, leading to an abundance of inflammatory cytokines such as TNF-α and IL-1. Moreover, Th17 cells drive RA-FLS to produce IL-6, IL-8, and matrix metalloproteinases, which potentiate structural damage [93]. A prominent role for Th17 cells has been demonstrated in bone destructive diseases such as RA and multiple myeloma [94, 95] (Figure 5).

### 3.4. B Cells

Multiple myeloma is a malignant B cell disease characterized by multiple bone lesions. These are caused by plasma cells expressing RANKL, which stimulate osteoclast formation, leading to osteolysis [96]. This phenomenon indicates that B cells could affect bone metabolism via RANKL expression. In RA, the pathophysiologic role of B cells is highlighted by the therapeutic success of B cell-depleting therapy with an anti-CD20 monoclonal antibody (rituximab) [97, 98]. B cells play an important role in producing autoantibodies. Although the role of autoantibodies such as rheumatoid factor (RF) and anti-citrullinated protein antibody is not fully understood, these autoantibodies are associated with more severe bone destruction [99]. Treatment with rituximab reduced bone destruction as well as joint inflammation. Taken together, these findings indicate that B cells contribute to bone destruction through RANKL expression and the production of autoantibodies in cooperation with other immune cells.

### 3.5. Neutrophils

The neutrophil is the most abundant type of white blood cell in mammals, and comprises an essential part of the innate immune system. Neutrophils normally circulate in the bloodstream and migrate to the site of inflammation in response to inflammatory stimuli. In the RA synovium, neutrophils regulate inflammation through the secretion of inflammatory mediators [100]. Histological analysis of bony lesions in humans and animal models indicates the involvement of neutrophils in pathogenic bone remodeling. Infiltration of neutrophils is observed in human periodontitis and experimental arthritis [101–103]. The RANKL-RANK-osteoprotegerin pathway is upregulated in activated neutrophils from inflammatory sites [104]. Membrane RANKL on neutrophils is strongly overexpressed after stimulation with lipopolysaccharide and thus mediates osteoclastic bone resorption through the interactions between neutrophils and osteoclasts [105]. The osteoclastogenic effect of neutrophils could be reproduced with purified neutrophil membranes, but not with culture supernatants from activated neutrophils. Thus, the effect of RANKL in activated neutrophils is predominantly mediated by the membrane-bound form, in contrast to activated T cells, where RANKL signaling is mediated by both cell surface and soluble RANKL [106, 107]. In addition, neutrophils affect the function of osteoblasts in children on chronic glucocorticoid therapy and in patients with tophaceous gout, resulting in altered bone remodeling [108, 109].

## 4. Conclusions

The human body attempts to maintain bone mass in order to maintain skeletal strength. Bone mass is not static but dynamic, and results from the formation or resorbtion of the bony matrix by osteoblasts or osteoclasts. In pathological states such as RA, in which bone resorption is favored over bone formation, osteoblasts are outnumbered by osteoclasts. Osteoclastogenesis is also favored over osteoblastogenesis by the inflammatory milieu. Recent studies have shown that numerous cytokines and immune cells have osteoclastogenic effects, although their exact roles in pathological states are difficult to determine because of the complexity of immune networks in the human body. Proinflammatory cytokines such as TNF-α, IL-1, IL-6, and IL-17 tend to be osteoclastogenic. The immune cells that participate in the pathogenesis of RA often enhance osteoclastogenesis by upregulating RANKL directly or by secreting proinflammatory cytokines that influence RANKL expression indirectly. Understanding the precise mechanisms of immune-mediated bone destruction would increase opportunities for target-specific inhibition of bone erosion or osteoporosis. Therapeutic interventions specifically targeting osteoclastogenesis might enable clinicians to spare bone mass in RA patients.

## Figures and Tables

**Table 1:** Roles of cytokines on osteoclastogenesis.

	*In Vitro *	*In Vivo *
TNF-α	Osteoclastogenic (i) Upregulates the expression of RANKL and osteoclast activators (ii) Enhances osteoclast differentiation synergistically with RANKL or independently of RANKL (iii) Inhibits osteoclast apoptosis	Osteoclastogenic (i) Upregulates the expression of RANKL and osteoclast activators (ii) Induces osteoclastogenesis in the presence or absence of RANKL (iii) Plays a critical role in inflammatory arthritis (iv) Associated with estrogen-deficient osteoporosis and joint destruction in RA
	References: [12, 21, 110–116]	References: [15, 24, 36, 117–119]

IL-1	Osteoclastogenic (i) Upregulates the expression of RANKL and osteoclast activators (ii) Enhances osteoclast differentiation synergistically with RANKL or independently of RANKL	Osteoclastogenic (i) Induces osteoclastogenesis in the presence or absence of RANKL (ii) Mediates TNF-*α*-induced osteoclastogenesis (iii) Participates in physiological bone metabolism (iv) Associated with estrogen-deficient osteoporosis
	References: [33, 35, 116, 120–122]	References: [23, 29–32, 36, 37]

IL-6	Osteoclastogenic (i) Upregulates the expression of RANKL and osteoclast activators (ii) Induces RANKL-dependent osteoclastogenesis	Osteoclastogenic (i) Enhances osteoclastogenesis in the prepubertal stage (ii) Supports osteoclastogenesis in callus formation during fracture healing (iii) Associated with bone loss from inflammatory arthritis and estrogen deficiency
	References: [10, 44, 123–129]	References: [46, 48, 49, 130–132]
	Antiosteoclastogenic (i) Suppresses the RANK signaling pathway (ii) Diverts cells into the macrophage lineage	Antiosteoclastogenic (i) Suppresses the differentiation of early osteoclast precursor cells (ii) Decreases osteoclast formation, leading to reduced bone turnover
	References: [6, 133, 134]	References: [46, 47, 135]

IL-17	Osteoclastogenic (i) Induces the expression of RANKL and proinflammatory cytokines (ii) Increases sensitivity to RANKL (iii) Enhances osteoclastogenesis via prostaglandin E2 (PGE2) in osteoblasts	Osteoclastogenic (i) Induces the expression of RANKL and proinflammatory cytokines (ii) Mediates estrogen-deficient osteoporosis
	References: [1, 53, 136–141]	References: [52, 142–144]
	Anti-osteoclastogenic (i) Suppresses osteoclast formation at high concentrations (ii) Inhibits osteoclastogenesis by induction of GM-CSF	
	References: [145, 146]	

IL-23	Osteoclastogenic Induces osteoclastogenesis via IL-17	Osteoclastogenic (i) Induces the expression of RANKL (ii) Expands myeloid-lineage osteoclast precursors
	References: [56]	References: [65, 147–150]
	Antiosteoclastogenic Inhibits osteoclast formation via T cells	Antiosteoclastogenic Limits the resorption of immature bone below the growth plate
	References: [57, 151]	References: [57]

**Table 2:** Effects of biologic therapies on bone.

	Mice	Human
TNF-*α* blockers	Bone-protective in inflammatory arthritis and estrogen deficiency	Bone-protective in inflammatory disease Changes in bone turnover markers in postmenopause (small observational study)
	References: [152–158]	References: [17, 19, 20, 159]

IL-1 blockers	Bone-protective in inflammatory arthritis and estrogen deficiency	Bone-protective in RA (not usually recommended; less effective than other biologic agents) Changes in bone turnover markers in postmenopause (small observational study)
	References: [28, 155, 157]	References: [19, 160, 161]

IL-6 blockers	Bone-protective in inflammatory arthritis No effects in estrogen deficiency	Bone-protective in RA
	References: [155, 156, 162, 163]	References: [41, 42, 164, 165]

IL-17 blockers	Bone-protective in inflammatory arthritis and estrogen deficiency	No data in bone metabolism
	References: [55, 138, 144, 166, 167]	

IL-23 blockers	Bone-protective in inflammatory arthritis	No data
	References: [56]	
